# A Pilot Crossover Study of the Tsubu-Tsubu Therapy Patch for Refractory Neuropathic Pain

**DOI:** 10.7759/cureus.117749

**Published:** 2026-10-10

**Authors:** Yusuke Senoo, Kyosuke Arakawa, Noriko Muto, Takashi Matsusaki, Ryuji Kaku, Hiroshi Morimatsu

**Affiliations:** 1 Department of Anesthesiology, Graduate School of Medicine, Dentistry and Pharmaceutical Sciences, Okayama University, Okayama, JPN; 2 Department of Medical Innovation, Graduate School of Medicine, Dentistry and Pharmaceutical Sciences, Okayama University, Okayama, JPN; 3 Department of Anesthesiology, Futaba Clinic, Okayama, JPN; 4 Department of Anesthesiology, Yamaguchi University Hospital, Ube, JPN; 5 Department of Anesthesiology, Mie University Hospital, Tsu, JPN

**Keywords:** complementary therapy, gate control theory of pain, neuropathic pain syndrome, pain management, transdermal patch

## Abstract

Background and objective

Nonpharmacological approaches for neuropathic pain are increasingly used, but noninvasive cutaneous stimulation devices remain insufficiently studied. The Tsubu-Tsubu Therapy Patch is an adhesive medical device containing glass beads and is suggested to provide analgesia through noninvasive stimulation based on the gate control theory. We conducted a prospective pilot clinical trial to explore its potential analgesic effect in patients with intractable neuropathic pain.

Methods

A crossover trial aimed to enroll 26 patients. Patients were randomized to receive the treatment or control patches during Days 1-8 and Days 15-22, with a washout period on Days 9-14. The primary outcome was the between-treatment difference in daily Visual Analog Scale (VAS) scores, analyzed using a linear mixed-effects model. Secondary outcomes included mean VAS differences, PainVision scores, sleep duration, PainDETECT, Pain Disability Assessment Scale (PDAS), Athens Insomnia Scale (AIS), and adverse events.

Results

Nine patients completed the study. Five had postherpetic neuralgia, and four had post-thoracotomy pain. The estimated treatment effect on daily VAS scores was -8.46 mm (95% CI, -11.83 to -5.09; p < 0.001). No significant effects of treatment period or day within the treatment period were observed. No statistically significant differences were observed in secondary outcomes, including mean VAS difference, sleep duration, pain-related measures (PainVision scores, PainDETECT, and PDAS), and AIS. Adverse events such as erythema, itching, and skin indentation occurred in both groups and were not serious.

Conclusions

This pilot trial found an estimated reduction in daily VAS scores with the Tsubu-Tsubu Therapy Patch compared with the control patch. Given the very small sample size and methodological limitations, these findings should be considered preliminary. Further adequately powered randomized controlled trials are needed to determine the clinical efficacy of this intervention.

## Introduction

Chronic neuropathic pain often causes severe suffering and disability, representing a major contributor to the global burden of disease. Its prevalence is estimated at approximately 6.9-10% in the general population. Treatment is frequently challenging, and many patients fail to achieve satisfactory pain relief with guideline-recommended first-line medications [1].

The Tsubu-Tsubu Therapy Patch^®^ (Medical Device Registration Number: 17B3X10007000001) is an adhesive sheet embedded with small glass beads that provides noninvasive stimulation when applied to painful areas. Although the proposed mechanism has been hypothesized to involve gate control theory, this has not been experimentally established [2].

In 1965, Melzack and Wall proposed the gate control theory, which posits that nonnociceptive inputs such as touch and pressure stimulate inhibitory interneurons in the spinal dorsal horn, thereby suppressing nociceptive neuronal activity and producing analgesia [3]. Based on this theory, various pain management approaches have since been developed. Common treatments that stimulate the skin or peripheral nerves include capsaicin, menthol, and transcutaneous electrical nerve stimulation (TENS), which have shown efficacy [4-7]. However, to our knowledge, no previous studies have evaluated adhesive therapies such as the Tsubu-Tsubu Therapy Patch for neuropathic pain. Because clinical evidence is lacking, exploratory clinical evaluation is needed. Both postherpetic neuralgia (PHN) and post-thoracotomy pain are forms of neuropathic pain [8,9]. To investigate the potential efficacy of the Tsubu-Tsubu Therapy Patch (hereafter referred to as the treatment patch) in patients with these intractable conditions, we conducted a prospective crossover intervention trial.

## Materials and methods

Participants

The study protocol was approved by the Institutional Review Board of Okayama University (approval no. 19-009) and was registered in the Japan Registry of Clinical Trials (jRCT1062200005) before patient enrollment.

This study was conducted at Okayama University from November 30, 2020, to September 5, 2022. Patients meeting the following criteria were included: (1) aged 20-90 years; (2) diagnosed with PHN, post-thoracotomy pain, or other neuropathic pain; (3) pain persisting for ≥6 months; and (4) pain localized to a single site that could be covered with up to 11 adhesive sheets. Pregnant patients and those with dermatological disorders were excluded.

Sample size

Based on previous studies, the standard deviation of the Visual Analog Scale (VAS) was estimated at 20 mm [10]. Therefore, a clinically meaningful improvement was defined as a 20-mm reduction in VAS scores. Assuming α = 0.05 and β = 0.2, a total of 24 cases were needed. Accounting for possible dropouts, a target of 26 cases was set. This assumption was used solely for sample size estimation and not as a responder definition for the primary analysis.

Study design

This study employed a single-blind design in which patients were blinded to patch allocation at the time of application. Randomization was performed using a sealed-envelope method. The preparation, allocation, and tabulation of the envelopes were conducted by a clinical research coordinator who was not involved in clinical practice or data analysis. The treatment and control patches were identical in appearance and adhesive properties and were visually indistinguishable at the time of application.

The 50 mm × 70 mm treatment patch has 1.2-mm-thick glass beads spaced 4.5 mm apart, covering an area of 25 mm × 42 mm. The control patch is an adhesive tape made of the same material and has the same dimensions as the treatment patch but does not contain glass beads. The treatment or control patches were applied by a trained physician or assistant three times per week. If a sheet became detached, patients were instructed to trim it and reapply it with reinforcement tape. During the first period, either the treatment or control patch was used for eight days. This was followed by a six-day washout period, after which the alternate patch was used for an additional eight days. The intervention used in this study consisted of physical stimulation with glass beads that lack pharmacological activity. The six-day washout period was selected to separate the two treatment periods and minimize potential residual sensory effects after patch removal. Although persistent analgesic, sensory, behavioral, or expectancy effects cannot be completely excluded, the intervention was nonpharmacological and did not involve sustained drug exposure.

The primary outcome was the between-treatment difference in daily VAS scores, analyzed using a linear mixed-effects model. Secondary outcomes included mean VAS differences, sleep duration, PainVision^®^ scores, PainDETECT, Pain Disability Assessment Scale (PDAS), and Athens Insomnia Scale (AIS). Sleep duration was assessed daily, whereas the other secondary endpoints were evaluated on the first and last days of each treatment period, for a total of four assessments.

The PainDETECT questionnaire assessed the presence of neuropathic pain components. It consists of nine items addressing pain characteristics and sensory symptoms, with total scores ranging from -1 to 38; higher scores indicate a greater likelihood of neuropathic pain [11]. This questionnaire was intended for screening rather than definitive diagnosis in this study. The PainVision system provided a quantitative evaluation of pain intensity through low-intensity electrical stimulation that does not itself induce pain. Pain scores are derived from the current perception threshold and the electrical current corresponding to pain-equivalent sensation [12]. The device used in this study was the PainVision PS-2100 N, manufactured by NIPRO Corporation (Settsu, Japan) and kept at the Pain Clinic Outpatient Department of Okayama University Hospital. The PDAS is a questionnaire used to evaluate how pain interferes with daily functioning. It consists of 20 items rated on a 0-3 scale, producing a total score between 0 and 60, with higher scores reflecting more severe disability [13].

Insomnia severity was assessed using the eight-item Japanese version of the Athens Insomnia Scale (AIS-J), with each item rated on a 4-point scale (0-3) [14,15].

Adverse events, nerve block use, and medication use were also recorded daily. Patients were instructed to record these pain-related items in a diary every night before going to bed. The timing of these interventions was also reviewed according to the treatment period.

Statistical analysis

Statistical analyses were performed using StataSE 18 (StataCorp LLC, College Station, TX, USA). Figures and some supplementary analyses were generated using EZR (R version 4.3.1, R Foundation for Statistical Computing, Vienna, Austria). Daily VAS scores and sleep duration were analyzed using linear mixed-effects models to account for repeated measurements within participants. Treatment, period, and day within each treatment period were included as fixed effects, and participant was included as a random intercept. The treatment effect was estimated as the difference between the Tsubu-Tsubu Therapy Patch and control conditions, with a 95% CI and p-values. For the primary outcome (VAS), treatment sequence was additionally included as a fixed effect in a sensitivity analysis to assess the robustness of the treatment effect to treatment order. Period effects were accounted for in the mixed-effects model, whereas carryover effects were not formally tested because of the small sample size. Wilcoxon’s signed-rank test was applied to paired or non-normally distributed data, including mean differences in VAS scores between the treatment and control patches and secondary outcomes (PainVision scores, PainDETECT, PDAS, and AIS). Adverse events were summarized descriptively. Statistical significance was defined as p < 0.05.

## Results

Participant flow

A total of 607 individuals were screened, of whom 598 were excluded for the following reasons: not meeting the inclusion criteria (n = 354), difficulty attending visits due to distance (n = 63), inability to apply the sheet to the target body sites (n = 34), existing skin disorders (n = 4), and other reasons (n = 143). As a result, nine patients were enrolled, and all nine completed both treatment periods. No data were missing. A Consolidated Standards of Reporting Trials (CONSORT)-compliant flowchart is presented in Figure 1.

**Figure 1:**
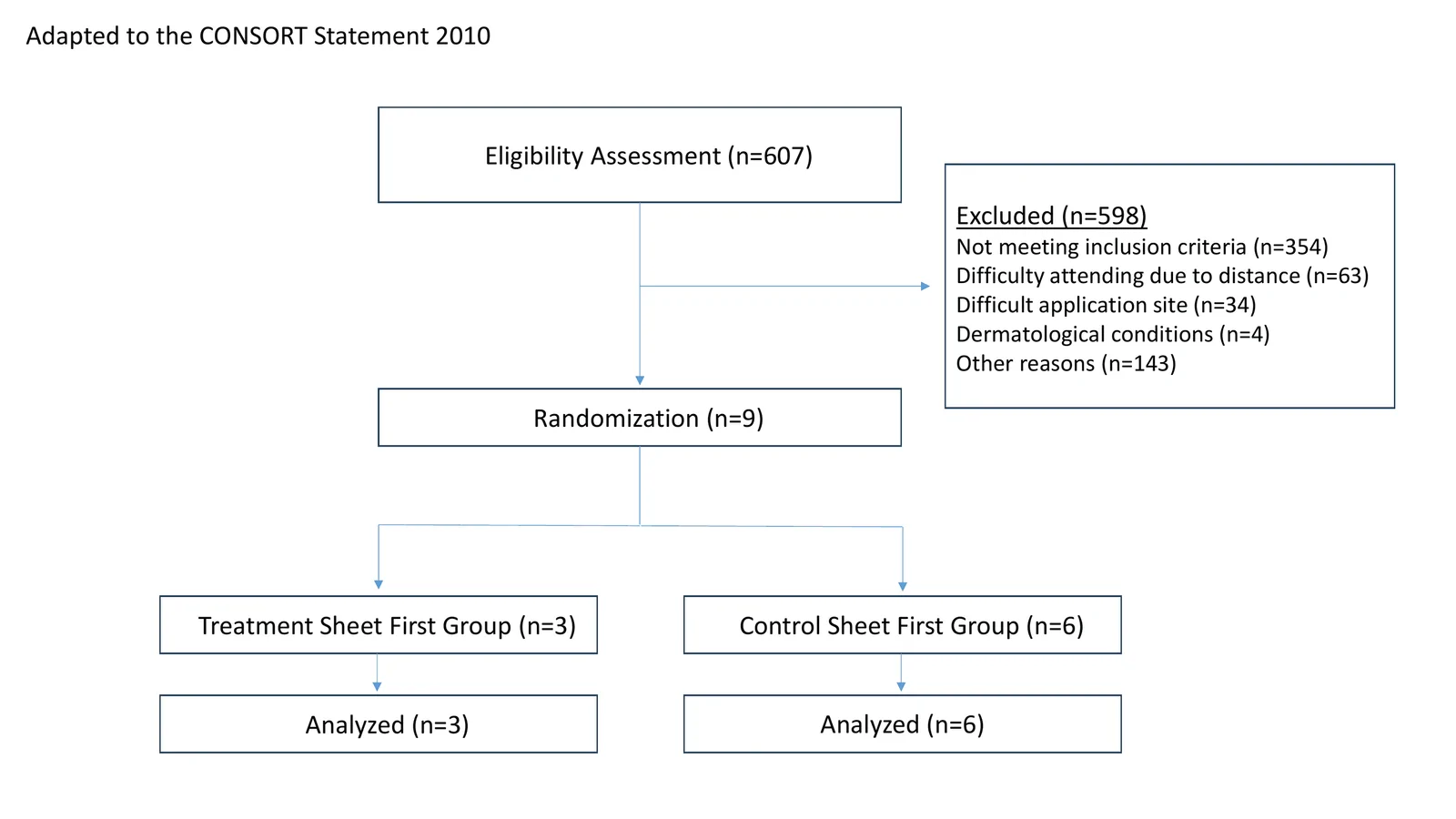
CONSORT flowchart of participant screening, exclusion, and enrollment. Nine patients were randomized and included in the analysis. CONSORT, Consolidated Standards of Reporting Trials

Patient characteristics

Table 1 summarizes the baseline characteristics. Nine patients were included, with a mean age of 67 ± 17.2 years and a mean baseline VAS score of 71.6 ± 10.0. The causes of pain were PHN (n = 5) and post-thoracotomy pain (n = 4).

**Table 1:** Baseline characteristics. Data are presented as mean ± SD or n (%). PHN, postherpetic neuralgia; VAS, Visual Analog Scale

Patient background (n = 9)	Mean ± SD or n (%)
Age (years)	67 ± 17.2
BMI (kg/m²)	24 ± 4.4
Baseline VAS scores	71.6 ± 10.0
Symptom duration	
6 months to <12 months	2 (22)
12 months to <24 months	3 (33)
≧24 months	4 (44)
Gender	
Male	6 (67)
Female	3 (33)
Cause of pain	
PHN	5 (56)
Postoperative pain	4 (44)
Medical history	
Hypertension	5 (56)
Hyperlipidemia	3 (33)
Diabetes mellitus	0
Medication	
Opioids	4 (44)
Neuropathic pain treatment	8 (88)
Nerve block therapy	1 (11)

Primary outcomes: mixed-effects model with VAS scores

Figure 2 shows changes in VAS scores over time. In the linear mixed-effects model, the estimated treatment effect on daily VAS scores was -8.46 mm (95% CI, -11.83 to -5.09; p < 0.001). There was no significant effect of treatment period (estimated difference, 0.17 mm; 95% CI, -3.20 to 3.54; p = 0.923) or day within the treatment period (estimated change, 0.11 mm/day; 95% CI, -0.58 to 0.81; p = 0.749). In the sensitivity analysis, which additionally adjusted for treatment sequence, the estimated treatment effect remained unchanged at -8.46 mm (95% CI, -11.83 to -5.09; p < 0.001). Treatment sequence was not statistically significant (estimated difference, -9.40 mm; 95% CI, -28.45 to 9.66; p = 0.334).

**Figure 2:**
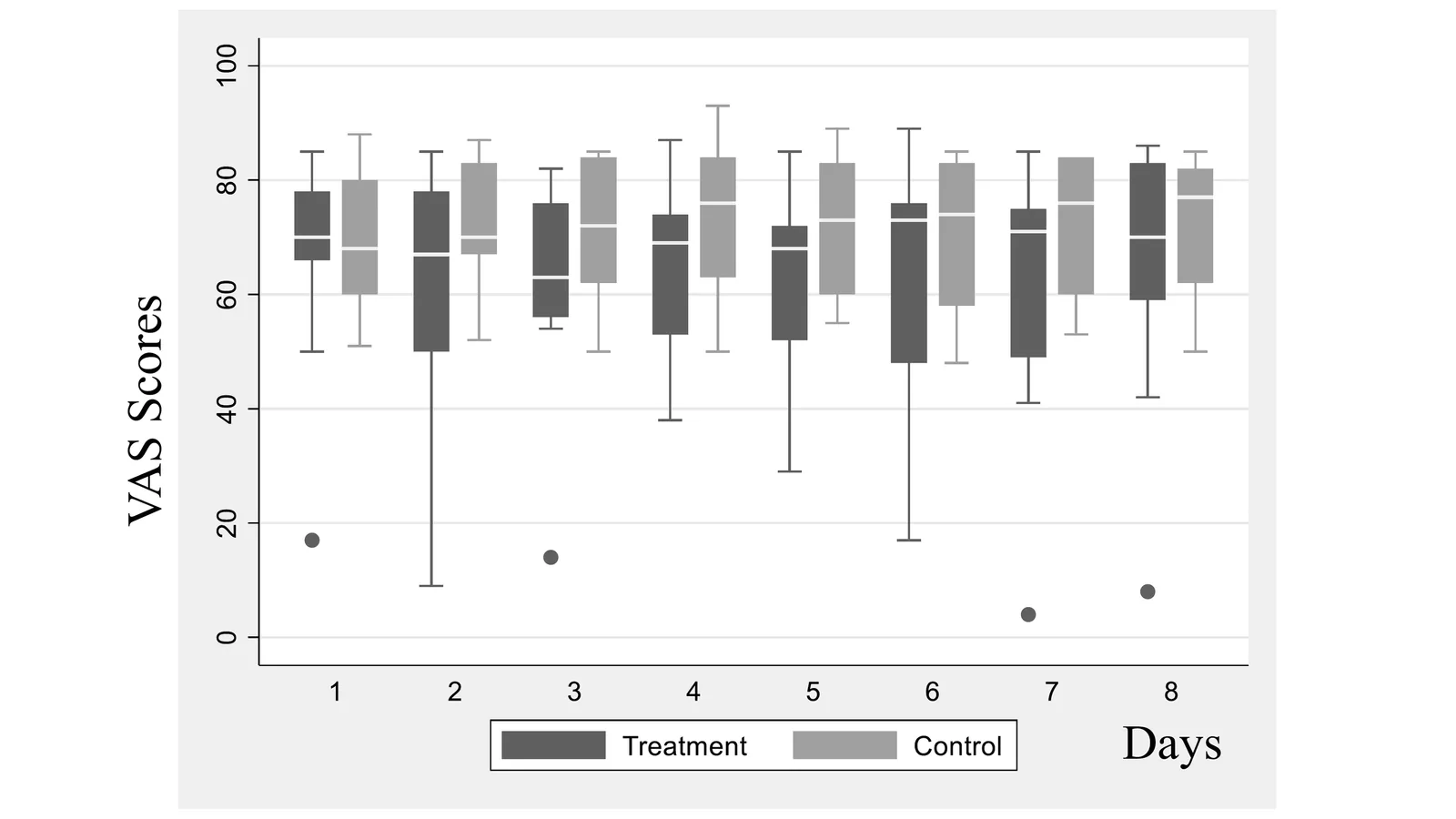
Box plots of VAS scores in the treatment and control groups over the study days. VAS, Visual Analog Scale

Secondary outcomes: mean differences in VAS scores, sleep duration, and other measures

When mean VAS scores were compared between conditions, the treatment patch was associated with lower scores than the control patch (mean difference, -8.04 mm; 95% CI, -17.6 to 1.46; p = 0.074). Figure 3 shows changes in sleep duration over time. Sleep duration was analyzed using the same linear mixed-effects model as the primary outcome. The estimated treatment effect was -24.4 min (95% CI, -51.9 to 3.0; p = 0.081). There was no significant effect of treatment period (estimated difference, 5.8 min; 95% CI, -21.7 to 33.3; p = 0.680) or day within the treatment period (estimated change, -0.55 min/day; 95% CI, -6.20 to 5.10; p = 0.849).

**Figure 3:**
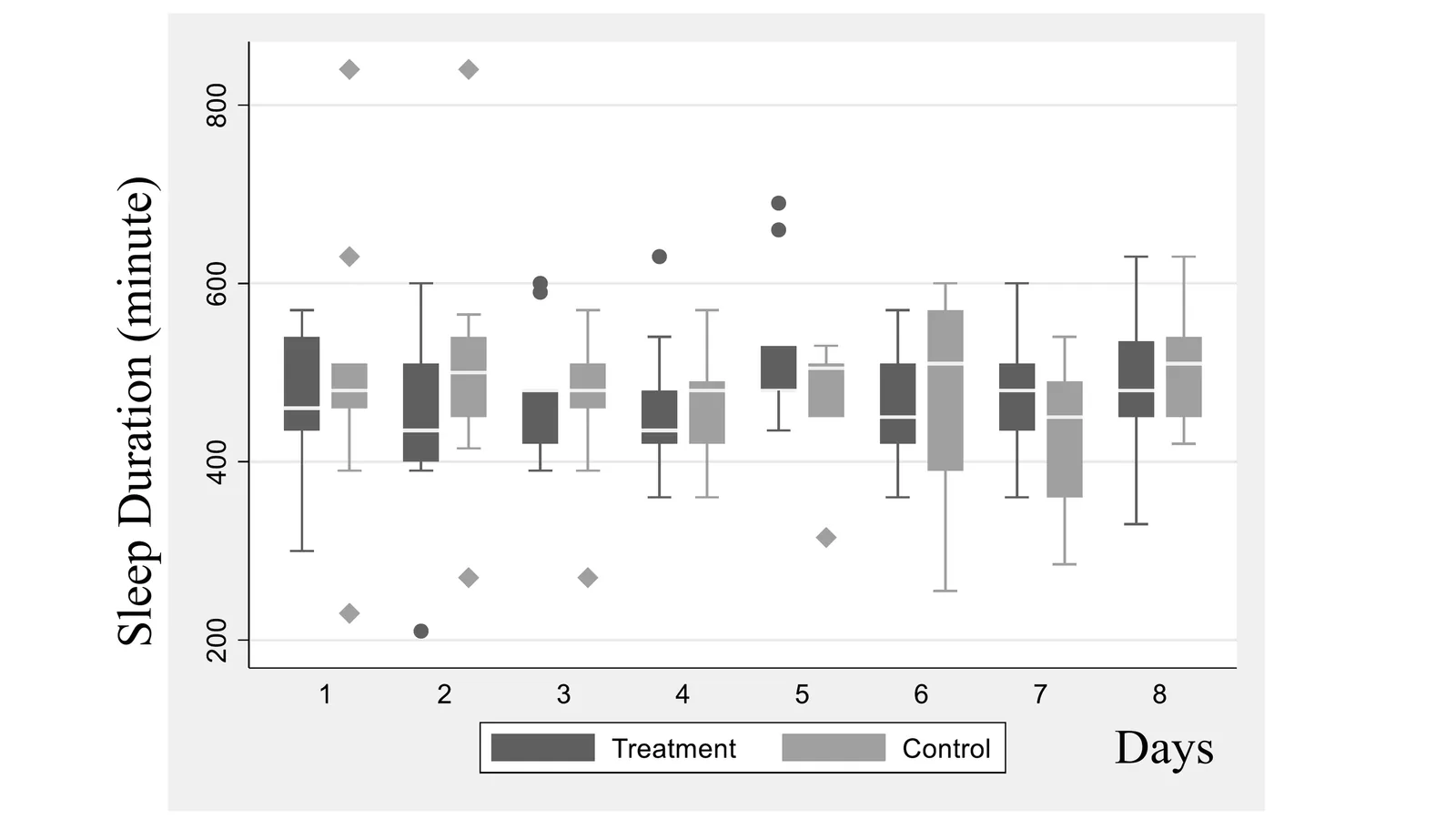
Box plots of sleep duration in the treatment and control groups over the study days.

Table 2 summarizes the other evaluation items; no statistically significant differences were observed.

**Table 2:** Other evaluation items. The mean changes in PainVision, PainDETECT, PDAS, and AIS were compared between the treatment and control periods. No statistically significant differences were observed. For all outcomes, negative changes indicate improvement. AIS, Athens Insomnia Scale; PDAS, Pain Disability Assessment Scale

Outcomes	Before the treatment patch	Before the control patch	Mean difference (95% CI)	p-value
PainVision	350 ± 292.0	509.8 ± 360.0	50.2 (-344 to 444.6)	0.44
PainDETECT	17.7 ± 3.5	16.4 ± 4.5	-1.0 (-6.3 to 4.3)	0.68
PDAS	19.6 ± 9.2	19.5 ± 8.5	0.44 (-7.1 to 8.0)	0.95
AIS	6.5 ± 3.1	6.5 ± 3.4	0 (-3.8 to 3.8)	0.81

Adverse events

The reported adverse events associated with the treatment and control patches were as follows: erythema (5 vs. 5 cases), blistering (3 vs. 3), pain at application (1 vs. 0), itching (5 vs. 3), insomnia (1 vs. 1), erosion (1 vs. 0), skin indentation (7 vs. 2), edema (0 vs. 1), nocturnal urinary frequency (0 vs. 1), and pain upon patch removal (3 vs. 2).

Other treatments

There were no changes in regularly prescribed analgesic medications in any patient during the study period. The total number of as-needed analgesic uses over the eight-day period was low in both groups, with 13 uses in the treatment group and nine uses in the control group. Nerve blocks were performed in only one of the nine patients: two trigger-point injections during the treatment patch period and one thoracic epidural block during the washout period. Given the very small number of nerve blocks, no statistical comparison was performed.

## Discussion

In this pilot crossover trial, VAS scores showed a downward trend during the treatment period. The linear mixed-effects model, which accounted for repeated measurements within patients, indicated a statistically significant treatment effect. Nevertheless, given the very small sample size and the exploratory nature of this study, the observed treatment effect should be interpreted cautiously. Further adequately powered randomized controlled trials are needed to confirm these findings and determine the clinical efficacy of the intervention. Recent advances in somatosensory neuroscience suggest that activation of mechanoreceptors can influence nociceptive processing through Aβ afferent pathways and spinal dorsal horn circuits, where tactile and pain-related inputs converge and interact [16]. In clinical practice, high-concentration capsaicin patches (8%) have demonstrated modest but statistically significant analgesic effects in peripheral neuropathic pain, with response rates of approximately 30-40% [3]. Topical menthol (1-10%) has provided transient pain relief in 40-60% of patients with localized neuropathic pain [4]. TENS has also shown variable efficacy, reducing pain intensity in 25-50% of patients with neuropathic pain [5,6].

Recent reviews indicate that the certainty of evidence for capsaicin patch therapy is low. In addition, the number of studies evaluating TENS remains too limited to permit reliable estimation of treatment effects, and neither treatment is currently strongly recommended [17]. Although there is no robust evidence that nonpharmacological treatments based on the gate control theory have a significant therapeutic effect in patients with neuropathic pain, some patients may still derive therapeutic benefit.

This study has several limitations that should be considered when interpreting the results. First, although a single-blind design was initially planned, it was difficult to maintain in practice. The adhesive patches occasionally became detached, requiring patients to reattach them. Consequently, blinding may have been compromised, potentially introducing measurement bias. Second, the number of patients was smaller than originally planned. In this study, patients were required to complete daily reports on pain and related factors. Among patients considered for enrollment, relatively few were able and willing to complete these daily pain diaries and were judged capable of providing reliable self-assessments, including because of cognitive limitations in some patients. Consequently, only nine eligible patients could be enrolled during the study period. Statistical power was reduced compared with the initial assumptions, and there is a possibility of failing to detect a significant difference, particularly when the effect size is small (type II error). Third, patients were instructed to record each episode of patch detachment. No detachment was observed in five of nine patients, while the remaining four patients experienced three to eight episodes of detachment and reapplication. The impact of these events is likely to be complex and could not be adequately assessed because of the small sample size. Therefore, a potential influence on the results cannot be excluded and should be considered a limitation. Fourth, the generalizability of the findings is limited by the small and clinically heterogeneous sample. The study included only nine patients, consisting of five patients with PHN and four with post-thoracotomy pain. Therefore, the findings should be considered preliminary and should not be generalized to the broader population of patients with neuropathic pain without confirmation in larger studies involving more diverse patient populations. Finally, both the treatment and control patches provided only transient cutaneous stimulation and sensory input without any pharmacological effects. The six-day washout period was intended to minimize potential residual sensory effects and provide separation between the two treatment periods. However, persistent analgesic, sensory, behavioral, or expectancy effects cannot be completely excluded.

Despite these limitations, the present study provides preliminary information that may assist in designing future adequately powered trials.

## Conclusions

This pilot crossover trial found an estimated reduction in daily VAS scores with the Tsubu-Tsubu Therapy Patch compared with the control patch. Given the very small sample size and methodological limitations, these findings should be considered preliminary. Further adequately powered randomized controlled trials are needed to determine the clinical efficacy of this intervention.
